# Full-Length Genome Sequence of Japanese Encephalitis Virus Strain FC792, Isolated from Guangxi, China

**DOI:** 10.1128/genomeA.01054-17

**Published:** 2017-11-30

**Authors:** Bingxia Lu, Yibin Qin, Bin Li, Ying He, Qunpeng Duan, Jiaxing Liang, Zhongwei Chen, Qianlian Su, Bingfen Bi, Wu Zhao

**Affiliations:** Guangxi Key Laboratory of Veterinary Biotechnology/Department of Virology, Guangxi Veterinary Research Institute, Nanning, Guangxi, China

## Abstract

We report here the complete genomic sequence of Japanese encephalitis virus (JEV) strain FC792, isolated from aborted fetuses of sows which were unimmunized with JEV vaccines in Guangxi Province, southern China. The complete JEV genome of strain FC792 had the highest nucleotide homology (99.7%) and amino acid identity (99.4%) with the sequence of JEV strain SA14-14-2 (GenBank accession number AF315119). Phylogenetic analysis showed that strain FC792 had the closest phylogenetic relationship to the sequence of strain YUNNAN0901 (GenBank accession number JQ086762). This study will help us understand the molecular pathogenesis and genetic diversity of genotype III Japanese encephalitis virus in pigs.

## GENOME ANNOUNCEMENT

Japanese encephalitis virus (JEV) is a member of the *Flavivirus* genus in the family *Flaviviridae*. It is an enveloped virus, with a positive-sense single-stranded RNA genome that contains a big open reading frame (ORF) flanked by 5′ and 3′ untranslated regions (UTRs) (1). The ORF encodes a polyprotein consisting of structural proteins (capsid [C], membrane [prM/M], and envelope [E]) and nonstructural proteins (NS1, NS2A, NS2B, NS3, NS4A, NS4B, and NS5) (2, 3). JEV infection can cause abortion or stillbirth in pregnant sows, piglets that are mummified or weak at birth, boar orchitis, etc., leading to great economic losses to pig-raising industries (4). JEV infection can also lead to acute encephalitis in humans; approximately 25 to 30% of JEV cases are fatal, and 50% result in permanent neuropsychiatric sequelae (5, 6).

In May 2016, JEV strain FC792 was isolated from aborted fetuses collected from a pig farm where pigs were administered JEV vaccines in Guangxi Province, southern China. The 5′ and 3′ ends of the viral genome of FC792 were confirmed by a SMARTer Rapid Amplification of cDNA Ends (RACE) cDNA amplification kit (Clontech, Japan). The other parts were generated by 10 overlapping cDNA fragments to encompass the entire genome and were determined by genome-walking sequencing. All PCR products were gel purified using the QIAquick gel extraction kit (Qiagen), cloned into the pMD18-T vector (TaKaRa), and then sequenced on a 3730 DNA analyzer (Applied Biosystems). The genome was assembled using the software program DNAStar (version 7.0).

The complete genome sequence of FC792 is 10,977 nucleotides (nt) in length [excluding the poly(A) tail] and includes a 5′ untranslated region (5′ UTR) (nt 1 to 95), structural proteins (capsid [C], nt 96 to 476; membrane [prM/M], nt 477 to 977; and envelope [E], nt 978 to 2477), nonstructural proteins (NS1, nt 2478 to 3713; NS2A, nt 3714 to 4214; NS2B, nt 4215 to 4607; NS3, nt 4608 to 6464; NS4A, nt 6465 to 6911; NS4B, nt 6912 to 7676; and NS5, nt 7677 to 10391), and a 3′ UTR (nt 10395 to 10977). Analysis of the full genome sequences demonstrates that the nucleotide sequence homologies between FC792 and the reported JEV full genome sequences vary from 84.6% to 99.7%, while the amino acid identities vary from 96.2% to 99.4%. Strain FC792 has the highest nucleotide homology (99.7%) and amino acid identity (99.4%) to the sequence of strain SA14-14-2 (GenBank accession number AF315119, genotype III). Phylogenetic analysis showed that JEV strain FC792 has the closest phylogenetic relationship to the sequence of strain YUNNAN0901 (GenBank accession number JQ086762, genotype III).

In recent years, JEV strains have been isolated from mummified fetuses or aborted fetuses of sows that have been reported in China with a high nucleotide homology (99.6% to 99.7%) to strain SA14-14-2, such as strain YUNNAN0901 and strain JEV/sw/GD/2008 (GenBank accession number KX965684). This is a very noteworthy problem. It suggests that the safety and immunological effects of JEV strain SA14-14-2 used as a vaccine for pigs need to be reassessed.

The genome sequence reported in this study will promote a better understanding of the molecular pathogenesis and genetic diversity of JEV in pigs.

### Accession number(s).

The complete genome sequence of JEV strain FC792 has been deposited in GenBank under the accession number MF002373.
